# Community Curation of Microbial Metabolites Enables Biological Insights of Metabolomics Data

**DOI:** 10.64898/2026.01.24.701521

**Published:** 2026-01-26

**Authors:** Helena Mannochio-Russo, Wilhan D. Gonçalves Nunes, Shipei Xing, Fernanda de Oliveira, Andrés Mauricio Caraballo-Rodríguez, Paulo Wender Portal Gomes, Vincent Charron-Lamoureux, Julius Agongo, Nicole E. Avalon, Tammy Bui, Lucia Cancelada, Marc G. Chevrette, Andrés Cumsille, Moysés B. de Araújo, Marilyn De Graeve, Victoria Deleray, Mohamed S. Donia, Mutsawashe B. Dzveta, Yasin El Abiead, Ronald J. Ellis, Donald Franklin, Neha Garg, Harsha Gouda, Claude Y. Hamany Djande, Anastasia Hiskia, Benjamin N. Ho, Chambers C. Hughes, Sunghoon Hwang, Sofia Iliakopoulou, Jennifer E. Iudicello, Alan K. Jarmusch, Triantafyllos Kaloudis, Irina Koester, Robert Konkel, Hector H. F. Koolen, Kine Eide Kvitne, Sabina Leanti La Rosa, Anny Lam, Santosh Lamichhane, Motseoa Lephatsi, Scott Letendre, Sarolt Magyari, Hanna Mazur-Marzec, Daniel McDonald, Ipsita Mohanty, Mónica Monge-Loría, David J. Moore, Thiago André Moura Veiga, Musiwalo S. Mulaudzi, Lerato Nephali, Griffith Nguyen, Martin Orságh, Abubaker Patan, Tomáš Pluskal, Phillip B. Pope, Lívia Soman de Medeiros, Paolo Stincone, Andrej Tekel, Sydney Thomas, Ralph R. Torres, Shirley M. Tsunoda, Fidele Tugizimana, Martijn van Faassen, Felipe Vasquez-Castro, Giovanni A. Vitale, Berenike C. Wagner, Crystal X. Wang, Sevasti-Kiriaki Zervou, Haoqi Nina Zhao, Simone Zuffa, Daniel Petras, Laura-Isobel McCall, Rob Knight, Mingxun Wang, Pieter C. Dorrestein

**Affiliations:** 1 Skaggs School of Pharmacy and Pharmaceutical Sciences, University of California San Diego, La Jolla, CA, USA; 2 Department of Biotechnology, Engineering School of Lorena, University of São Paulo, Lorena, São Paulo State, Brazil; 3 Amazon Integrated Metabolomics Center (CIMAZON), Institute of Natural and Exact Sciences, Federal University of Pará, Rua Augusto Corrêa, 01 - Guamá 66075-110, Belém, PA, Brazil; 4 Department of Pharmaceutical Sciences, School of Pharmacy & Pharmaceutical Sciences, University of California, Irvine, Irvine, CA, USA; 5 Robert A; 6 Center for Marine Biotechnology and Biomedicine, Scripps Institution of Oceanography, University of California, San Diego, La Jolla, CA, USA; 7 Scripps Institution of Oceanography, University of California, San Diego, La Jolla, CA, USA; 8 Department of Chemistry and Biochemistry, University of California, San Diego, La Jolla, CA, USA; 9 Department of Plant Pathology, University of Wisconsin-Madison, Madison, WI 53706, USA; 10 Wisconsin Institute for Discovery, University of Wisconsin-Madison, Madison, WI 53706, USA; 11 Grupo de Pesquisa em Metabolômica e Espectrometria de Massas, Universidade do Estado do Amazonas, 69065-001 Manaus-AM, Brazil; 12 Instituto de Ciências Exatas e Tecnologia, Universidade Federal do Amazonas, 69103-128 Itacoatiara-AM, Brazil; 13 Laboratory of Integrative Metabolomics, Department of Translational Physiology, Infectiology and Public Health, Ghent University, Salisburylaan 133, 9820 Merelbeke, Belgium; 14 Department of Molecular Biology, Princeton University, Princeton, NJ, USA; 15 Research Centre for Plant Metabolomics, Department of Biochemistry, University of Johannesburg, Johannesburg, South Africa; 16 Department of Neurosciences, University of California San Diego, San Diego, CA 92093, USA; 17 HIV Neurobehavioral Research Program, University of California San Diego, San Diego, CA 92093, USA; 18 Department of Psychiatry, University of California San Diego, San Diego, CA 92093, USA; 19 School of Chemistry and Biochemistry, Georgia Institute of Technology, Atlanta, Georgia 30332, United States; 20 Center for Microbial Dynamics and Infection, Georgia Institute of Technology, Atlanta, Georgia 30332, United States; 21 Institute of Nanoscience & Nanotechnology, NCSR Demokritos, Athens, Greece; 22 Department of Microbial Bioactive Compounds, Interfaculty Institute of Microbiology and Infection Medicine (IMIT), University of Tübingen, 72076 Tübingen, Germany; 23 Cluster of Excellence EXC 2124: Controlling Microbes to Fight Infection, University of Tübingen, 72076 Tübingen, Germany; 24 German Center for Infection Research (DZIF), Partner Site Tübingen, 72706 Tübingen, Germany; 25 AquOmixLab, Department of Water Quality Control, Athens Water Supply & Sewerage Company (EYDAP SA), Athens, Greece; 26 National Institute of Environmental Health Sciences, National Institutes of Health, Research Triangle Park, NC, USA; 27 Woods Hole Oceanographic Institution, Woods Hole, MA, USA; 28 Department of Marine Biology and Biotechnology, Faculty of Oceanography and Geography, University of Gdańsk, Gdynia, Poland; 29 Faculty of Chemistry, Biotechnology and Food Science, Norwegian University of Life Sciences, 1432 Ås, Norway; 30 Institute of Biomedicine, Faculty of Medicine, & Turku Clinical Microbiome Bank, Clinical Microbiology & Microbe Centre, Turku University Hospital and University of Turku and Wellbeing Services County of Southwest Finland,Turku, Finland; 31 Department of Medicine, University of California San Diego, La Jolla, CA, USA; 32 Department of Chemistry, Simon Fraser University, 8888 University Drive,Burnaby, Canada; 33 Department of Pediatrics, University of California San Diego, La Jolla, CA, USA; 34 Institute of Environmental, Chemical and Pharmaceutical Sciences, Department of Chemistry, Federal University of São Paulo, Diadema, 09972-270, Brazil; 35 Department of Biochemistry, University of Johannesburg, Johannesburg, South Africa; 36 Institute of Organic Chemistry and Biochemistry of the Czech Academy of Sciences, Prague, Czech Republic; 37 Department of Physical and Macromolecular Chemistry, Faculty of Science, Charles University, Albertov 6, 120 00 Prague 2, Czech Republic; 38 The Centre for Microbiome Research, Queensland University of Technology, 4102, Woolloongabba, Australia; 39 University of Tübingen, Interfaculty Institute of Microbiology and Infection Medicine, Tübingen, Germany; 40 Department of Laboratory Medicine, University of Groningen, University Medical Center Groningen, 9713 GZ Groningen, the Netherlands; 41 Department of Biochemistry, University of California Riverside, Riverside, CA, USA; 42 Department of Chemistry and Biochemistry, San Diego State University, San Diego, California, USA; 43 Center for Microbiome Innovation, University of California San Diego, La Jolla, CA, USA; 44 Department of Computer Science and Engineering, University of California San Diego, La Jolla, CA, USA; 45 Shu Chien-Gene Lay Department of Bioengineering, University of California San Diego, La Jolla, CA, USA; 46 Halıcıoğlu Data Science Institute, University of California San Diego, La Jolla, CA, USA; 47 Hong Kong University of Science and Technology Jockey Club Institute for Advanced Study, Hong Kong SAR, China; 48 Department of Computer Science and Engineering, University of California Riverside, Riverside, CA, USA; 49 Center for Microbiome Innovation, University of California, San Diego, La Jolla, CA, 92093, USA; 50 Collaborative Mass Spectrometry Innovation Center, Skaggs School of Pharmacy and Pharmaceutical Sciences, University of California San Diego, La Jolla, CA, USA; 51 Department of Pharmacology, University of California San Diego, La Jolla, CA, 92093, USA

## Abstract

Microbial metabolites play a critical role in regulating ecosystems, including the human body and its microbiota. However, understanding the physiologically relevant role of these molecules, especially through liquid chromatography tandem mass spectrometry (LC-MS/MS)-based untargeted metabolomics, poses significant challenges and often requires manual parsing of a large amount of literature, databases, and webpages. To address this gap, we established the Collaborative Microbial Metabolite Center knowledgebase (CMMC-KB), a platform that fosters collaborative efforts within the scientific community to curate knowledge about microbial metabolites. The CMMC-KB aims to collect comprehensive information about microbial molecules originating from microbial biosynthesis, drug metabolism, exposure-related molecules, food, host-derived molecules, and, whenever available, their known activities. Molecules from other sources, including host-produced, dietary, and pharmaceutical compounds, are also included. By enabling direct integration of this knowledgebase with downstream analytical tools, including molecular networking, we can deepen insights into microbiota and their metabolites, ultimately advancing our understanding of microbial ecosystems.

## Introduction:

Of the thousands of molecules detectable by liquid chromatography-mass spectrometry (LC-MS/MS) in typical biospecimens, the host-associated microbiome modifies 15–70% of them depending on the specific organ or biofluid analyzed^1^. In a typical untargeted metabolomics profile from humans, only about 10% of the acquired spectra can be annotated, and among these, an even smaller portion can be directly traced to microbial origins. Humans have three major sources of microbial metabolites: 1) microbial metabolism of host-derived metabolites^2^; 2) microbial metabolism of molecules from food and beverages^3^; and 3) microbial metabolites assembled *de novo* using proteins encoded by genetic elements often arranged as gene clusters (in bacteria, archaea, fungi, and, recently, discovered to be widespread in phages)^4^. Additionally, microbial metabolites found in humans originate from microbial processing of xenobiotics other than food, such as plasticizers, pollutants, medications, and environmental molecules absorbed through the skin or inhaled ^5–7^.

Despite the critical importance of microbiome-derived metabolites to human health – including those involved in the microbe-gut-brain^8^ and microbe-diet-host axes^9^ – and other ecosystems, there is no centralized knowledgebase where the scientific community can deposit, curate, access, and reuse that knowledge. Existing resources have assessed how the microbiome influences the consumption and production of about 900 largely primary microbial metabolites^10^, and have compiled literature-curated information about 3,269 microbiome-derived metabolites^11^, but most of these metabolites are not unique to microorganisms or have been curated from metabolic models, which tend to capture mainly primary metabolism rather than specialized metabolites which can be more biologically relevant for host-microbiome interactions^12,13^. In addition, some targeted commercial metabolite platforms claim to capture up to ~140 microbial molecules, but many of those could also come from diet or the host, highlighting the challenge in the field with accurately understanding microbiome-derived metabolites^14^. microbeMASST, our recent tool that enables searching a fragmentation spectra against a reference microbial metabolomics database, allows direct connection between bacteria and fungi and microbially-derived molecules they produce^15^. However, microbial metabolites that have been recently discovered (or even yet to be discovered), the organisms and the genes responsible for their production, and/or their related activities, are not yet systematically cataloged. Therefore, reusing this information is a bottleneck for the community that aims to mechanistically understand the microbiome.

To complement existing microbial metabolite resources, as well as to enable annotation of structurally uncharacterized metabolites (captured as MS/MS spectra), we have created the Collaborative Microbial Metabolite Center knowledgebase (CMMC-KB). Leveraging the Global Natural Product Social Molecular Networking (GNPS)^16^ mass spectrometry analysis ecosystem, the CMMC-KB enables collaboration within the scientific community to curate knowledge on microbial metabolites or metabolites that might influence microbial metabolites (drugs, food, etc). The goal of this initiative is to facilitate biological interpretations of microbiome-derived molecules. With downstream molecular networking integration, the CMMC-KB allows users to visualize MS/MS spectra of compounds classified as microbial metabolites within molecular networks (grouped by MS/MS spectral similarity), even if their structures remain unknown. Furthermore, it provides information on microbial producers, the sources of the molecules, associated genes or sequences, and their biological activities, if known. For a broader investigation of the metabolome, molecules from other sources, such as endogenous molecules, compounds ingested through diet, and drugs, among others, are also included as part of this resource. The CMMC-KB is a user-accessible, collaboratively curated, and continuously evolving microbiome resource. Further, to encourage data deposition, we offer web-based analysis tools, including accessible web applications, that benefit both the data contributors and the broader community. In alignment with the FAIR data principles, we are committed to building this central knowledge hub in collaboration with the scientific community.

## Results and discussion:

**Figure 1.**
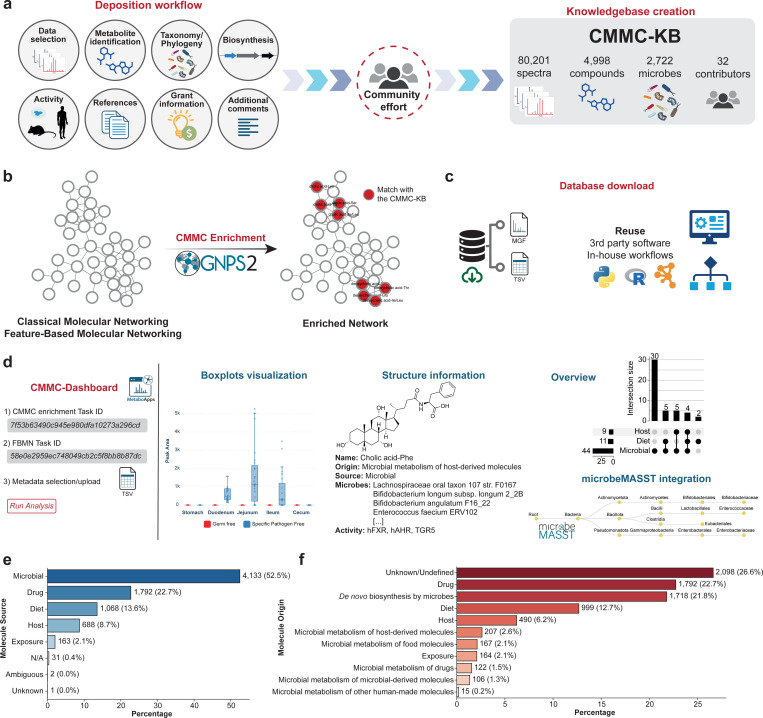
Overview of the CMMC-KB capabilities and depositions. **(a)** Inputs accepted for community depositions and current numbers as of December 2025. **(b)** CMMC enrichment workflow in GNPS2, which annotates molecular networks (generated from Classical or Feature-Based Molecular Networking^25,26^) by matching experimental spectra to the CMMC-KB and retrieving associated metadata. **(c)** Download options as MGF and TSV files, enabling reuse in third-party software and in-house workflows. **(d)** The CMMC-Dashboard is a web application that enables users to utilize outputs from the FBMN and CMMC enrichment workflows, along with uploaded metadata, to generate visualizations for exploring matches to the CMMC-KB (e.g., boxplots for statistical evaluation, structure cards, UpSet-style overviews, and microbeMASST integration). Distribution of the deposited compounds (December 2025) by **(e)** molecule source and **(f)** molecule origin. Icons were obtained from Bioicons.com.

The CMMC-KB (https://cmmc-kb.gnps2.org/) is a knowledgebase derived from contributions by the scientific community and comprises spectral (MS/MS data) and structural (chemical structures) information about microbially-derived compounds, as well as dietary, host-derived, and other exposure-related compounds. Contributions to the CMMC-KB are facilitated through a dedicated workflow in GNPS2 (a second major implementation of the GNPS ecosystem), enabling users to upload information organized into eight main sections: 1) MS/MS data selection, 2) metabolite identification, 3) taxonomy/phylogeny selection, 4) biosynthesis, 5) activity, 6) references, 7) funding information, and 8) additional comments (Figure 1a). The community can contribute to this resource by uploading knowledge for a single molecule at a time or in batches of molecules. A comprehensive documentation page is available to guide users on the recommended inputs (https://cmmc.gnps2.org/deposition_documentation/). While MS/MS spectra are recommended, they are not required, and users may deposit the molecular structure. Additionally, the molecules deposited can be classified as confirmed (e.g., observed experimentally in microbial cultures^17^, observed in colonized but not in germ-free mice, etc.) or predicted to be microbial (e.g., MS/MS of synthetic compounds with matches against other microbial resources like microbeMASST^15^). As of December 2025, the knowledgebase comprises 80,201 MS/MS spectra from 4,998 compounds that were linked to 2,722 microorganisms. These numbers reflect the collective efforts of more than 30 researchers who have contributed to the development of this resource to date^17–21^. Among the compounds deposited, their molecular sources were mainly classified as microbial, drug, or diet-related (Figure 1b). The majority of compounds had a known molecular origin, such as drugs, *de novo* biosynthesized by microbes, or diet, with 25.9% classified as unknown/undefined (Figure 1c). Since diet, drugs, and host-derived molecules can act as confounders, and because they often influence microbial metabolite production, the CMMC-KB includes and annotates these non-microbial compounds within a single, comprehensive resource.

To facilitate the use of information deposited in the CMMC-KB, there are three ways to access and leverage the knowledgebase. First, data are available for direct download in TSV/CSV and MGF formats from the website, allowing integration into customized in-house or third-party workflows. Second, we developed a workflow within the GNPS2 ecosystem that enables downstream enrichment of molecular networks (CMMC enrichment) with information from the CMMC-KB. Finally, we created an interactive web application^22^, CMMC-Dashboard (https://cmmc-dashboard.gnps2.org/), which allows users to visually explore and interpret the data in an accessible and user-friendly manner.

Many compounds deposited as microbial metabolites may also come from other sources. For example, some amino acids and fatty acids can be synthesized by microorganisms, ingested through diet, and also produced by host cells. To address this issue, we refined source annotations in the CMMC-KB by reanalyzing four datasets available in the public domain which contained tissues or biofluids of germ-free (GF) and colonized mice (MSV000079949^1^, MSV000088040^23^, MSV000097485, MSV000090974^24^), also considering mouse diet (chow) for metabolomics data, when available. We ran feature-based molecular networking (FBMN)^25^ followed by CMMC enrichment in GNPS2. Entries initially labelled as “microbial” were selected in the CMMC-Dashboard, and boxplots were plotted for GF vs. colonized mice (and also vs. diet, if available). We defined a metabolite as “microbial-only” when it was absent in the GF group but present in colonized mice (Supplementary Figure S2a-c), and added labels as “diet” and/or “host” when the metabolite was detected in GF and/or chow. This classification may include both microbially-produced metabolites and microbe-induced host metabolites, which cannot be distinguished without additional experimental validation. This targeted curation expanded the information available in the CMMC-KB by providing additional classifications for 88 metabolites (1.76% of the compounds deposited).

**Figure 2.**
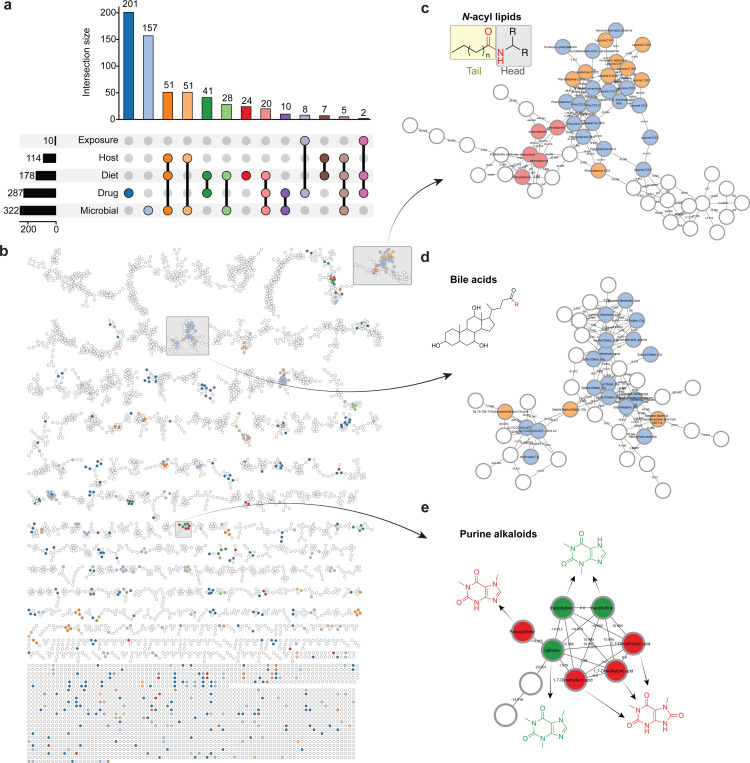
Application of CMMC-KB enrichment to fecal metabolomics data from the American Gut Project. **(a)** Source distribution of metabolites matched to the CMMC-KB from a subset of the American Gut Project (n = 1,993 samples)^27^. The UpSet plot was generated using the CMMC-Dashboard. **(b)** Molecular network visualization with nodes colored by metabolite source annotation from the CMMC-KB. Each node represents a unique mass spectral feature, and edges connect features with similar MS/MS spectra (cosine similarity threshold set to 0.5). **(c-e)** Zoomed-in views of selected molecular networks with distinct source annotations. These subnetworks illustrate the tool’s capability to rapidly identify and visualize structurally related compounds sharing common sources within complex metabolomic datasets. The colors of the nodes in **b-e** match the upset plot colors in **a**.

To illustrate how the CMMC-KB can benefit researchers, we used this resource to investigate microbial metabolites in a subset of the American Gut Project (n = 1,993 files), a citizen-science cohort with participation open to the general population (primarily from the United States, the United Kingdom, and Australia)^27^. In this example, FBMN was performed, followed by CMMC enrichment to annotate features based on spectral matches to the knowledgebase. The source distribution of matched metabolites revealed a diverse chemical landscape, with compounds classified across multiple categories, including microbial, host-derived, and xenobiotic sources (Figure 2a). By overlaying this information onto the molecular network, one can rapidly visualize regions enriched in specific source categories (Figure 2b). This network-based visualization facilitates hypothesis generation by revealing which networks of structurally related compounds share common sources. Zooming into specific network regions (Figure 2c-e) demonstrates the utility of the tool for detailed exploration of individual molecular families, where users can have an integrated view of the source annotations, structural relationships, and associated metadata for compounds of interest. With such an overview, users can target the investigation of specific classes of compounds with important biological functions. For instance, microbially-derived bile acids play crucial roles in immune regulation,^28^ and have been implicated in conditions ranging from inflammatory bowel disease to metabolic disorders and neurocognitive function^29,30^. Similarly, *N*-acyl lipids serve as signaling molecules involved in immune homeostasis, energy metabolism, and gut-brain axis communication^31,32^. The ability to identify and annotate these metabolite families (along with their potential microbial, dietary, or host origins) enables researchers to formulate targeted hypotheses about microbiome-host interactions and prioritize investigations into specific microbial producers, dietary influences, or disease associations. This analysis exemplifies how the CMMC-KB, combined with molecular networking, provides an efficient workflow to survey complex metabolomic datasets and identify features warranting further mechanistic investigation. Importantly, while the biological roles of bile acids and *N-*acyl lipids in gut-microbiome interactions were previously established, the CMMC-KB workflow enabled their rapid annotation and source classification in the American Gut Project cohort – a process that would have required extensive manual literature curation. This cross-cohort validation demonstrates that known metabolite-microbiome relationships can be efficiently detected across diverse population studies using this framework.

Beyond this specific use case, the CMMC-KB has been applied to diverse biological contexts that demonstrate its versatility in addressing complex research questions, ranging from the human microbiome, natural products, and environmental fields (Supplementary Material). In clinical settings, this resource enabled mapping drug metabolism across multiple biofluids in people with HIV, revealing that while antiretrovirals like ritonavir undergo extensive microbial transformation in the gut, these derivatives remain largely absent from plasma and cerebrospinal fluid (Supplementary Figure S1). Comparisons between germ-free and colonized mice facilitated the annotation and the refinement of microbial metabolites, including bile acid conjugates and *N*-acyl lipids, illustrating the dynamic, community-driven nature of the knowledgebase as new data emerge (Supplementary Figure S2). Environmental applications include the detection of bioactive cyanobacterial metabolites in Lake Marathon water samples, providing actionable information for water safety management (Supplementary Figure S3). In disease contexts, the tool identified a microbiome-derived bile acid conjugate altered by *Leishmania* infection in hamster tissues, linking microbial metabolism to parasite-induced disturbances (Supplementary Figure S4). Finally, in coral holobiont research, the CMMC-KB successfully disentangled bacterial versus zooxanthellae metabolic contributions in synthetic communities, revealing siderophore-mediated interactions that would have been difficult to assign using traditional approaches alone (Supplementary Figure S5).

When using the CMMC-KB, users should be aware of two key limitations. First, spectral matches are based on cosine similarity^33^ or modified cosine similarity^26^, which cannot easily distinguish isomers that share very similar MS/MS patterns. As a result, isomeric compounds, including those originating from different sources, may have spectra with a high cosine similarity (e.g., deoxycholic acid is a microbial metabolite, chenodeoxycholic acid is host-derived, and their MS/MS cosine similarity is >0.9; Supplementary Figure S2d). Consequently, users may obtain spectral matches to metabolites of incorrect biological origin, which highlights the need for follow up experiments and analyses for validation. Whenever possible, users should acquire orthogonal data (e.g. UV-vis, retention time, ion mobility collision cross section (CCS)) obtained from authentic chemical standards for confirmation. Second, the microbial origin of metabolites also requires additional experimental validation beyond spectral matching. Users can employ complementary approaches such as pure culture studies, co-culture experiments with isotope tracing (e.g., ^13^C-labeled substrates), comparisons between germ-free and colonized animal models, or spatial metabolomics to confirm not only the accuracy of the annotation but also its microbial biosynthesis or transformation of the detected compounds. As entries and curated knowledge continue to grow with future studies and depositions, the CMMC-KB will increasingly empower researchers to gain biological insights on the role of the microbiome in human health and diverse ecosystems.

## Methods:

### CMMC-KB development

The CMMC knowledge portal was developed using the FAIR (Findable, Accessible, Interoperable, and Reusable) principles as a guideline^34^. It incorporates a series of Python workflows designed to process deposition files and generate visualization tables for all deposited information (**F**indable). In addition, the CMMC-KB server compiles the files required for molecular networking enrichment workflows, including the MGF for the spectral database and structural information for deposited metabolites (**A**ccessible). The KB server provides programmatic access through API endpoints (**I**nteroperable) to download the database files, enabling seamless integration and reuse of information within custom workflows (**R**eusable). The database is automatically compiled daily to ensure the workflows use the most up-to-date information available in the KB.

Each compound with an associated structure in the CMMC-KB is assigned a unique URL, enabling seamless cross-linking to external resources such as NPAtlas.^35,36^ The structure page provides users with tools to explore the molecular structure of metabolites and access all available information for a given molecule and mass spectra available in the knowledgebase. From this interface, users can also contribute additional data by being redirected through a URL to a prepopulated deposition page containing the USI, molecule name, and SMILES/InChI, where further information can be added.

The CMMC-KB Statistics page is a public, daily refreshed summary of the knowledgebase that reports coverage (total unique mass spectra), composition (distributions by metabolite source/origin), and temporal dynamics (new deposits over time), alongside contributor activity.

### CMMC-KB deposition workflow

The CMMC-KB deposition workflow is implemented as a Nextflow-based pipeline^37^ on GNPS2, which runs a series of Python scripts to validate and process user submissions. The deposition workflow supports both single (one molecule) and batch (multiple entries) deposition modes. In single-deposition mode, parameters are provided through a workflow form or YAML file, while in batch depositions, the input is provided through a TSV file. Each entry is checked against controlled vocabularies (e.g., source and origin) and must include valid spectral and structural identifiers: spectra are verified via the Metabolomics Spectrum Resolver API^38^ using USIs, and chemical structures (SMILES or InChI) are validated with the GNPS2 ChemicalStructureWebService API. Following validation, all data is submitted to the CMMC-KB server via POST requests. The necessary templates, including the TSV file and deposition instructions, are fully documented and available at https://cmmc.gnps2.org/deposition_documentation/.

### Network Enrichment workflow

The enrichment workflow is implemented as a Nextflow pipeline available within the GNPS2 ecosystem, and can be launched as a downstream analysis from the Classical or Feature-Based Molecular Networking results. This design enables users to annotate molecular networks with microbial information through a single-click integration. The workflow retrieves molecular networking outputs from both GNPS1 and GNPS2 jobs, including the network (.graphml) and associated spectral (.MGF) files. The retrieved spectra will be matched against the ones available in the CMMC-KB by cosine similarity, and the matches are further enriched with additional metadata if available, including microbial producers, taxonomy, chemical structure, biosynthetic gene clusters, molecular origin, activities, and compound classifications predicted using NPClassifier.^39^ The outputs include a library match TSV table and a new .graphml file with overlaid compound metadata information from the CMMC-KB matches. Additional visualizations, such as the producer lineage and taxonomic distribution, are generated from the NCBI Taxonomy IDs linked to each deposited spectrum. A documentation of the network enrichment workflow is available at https://cmmc.gnps2.org/network_enrichment/.

### CMMC-Dashboard web application

The CMMC Analysis Dashboard (https://cmmc-dashboard.gnps2.org/) was implemented as a web application using the Streamlit Python package to provide interactive access to results from the CMMC-KB enrichment workflow in combination with the FBMN data. The dashboard integrates directly with GNPS2 through Task IDs provided by the user. Task IDs from enrichment and FBMN workflows allow the application to fetch processed files, including enrichment results, FBMN quantification tables, molecular networks, and associated metadata. The dashboard can also be launched directly as a downstream analysis from the enrichment workflow results page, from which the required inputs will be prepopulated in the dashboard interface. A complete documentation for this tool can be found at https://wang-bioinformatics-lab.github.io/GNPS2_Documentation/metaboapp_CMMC_dashboard/.

After the inputs are specified, the dashboard merges enrichment outputs with quantification tables and metadata for downstream analyses. Statistical functionality includes the generation of box plots to compare metabolite abundances across groups, with options for stratification and multiple statistical tests. Overlaps of metabolite sources or origins can be visualized using UpSet plots^40^ derived from the enrichment results. Molecular network exploration is supported through interactive Plotly visualizations that highlight selected features within networks, incorporate delta-mass annotations for network edges, and enable export of figures. The dashboard further integrates microbeMASST^15^, allowing users to perform spectral searches based on a Universal Spectrum Identifier (USI) or feature ID, returning exact or analog matches with compounds from microbial cultures. This allows for taxonomically informed results with corresponding downloadable taxonomic trees.

## Supplementary Material

Supplement 1

## Data Availability

All the datasets used in this work as use cases of the CMMC-KB are available in MassIVE (massive.ucsd.edu). Raw data files from the American Gut Project, used in Figure 2, are deposited at MSV000080673. The feature finding step was performed in MZmine3, following the previous parameters used for this dataset^19^. Feature-Based Molecular Networking analysis and CMMC enrichment analysis for the American Gut Project use case can be found at https://gnps2.org/status?task=553c08a0e2274572a4edd2ba2d669668 and https://gnps2.org/status?task=2ac40effdb0f404fa6a045a580ff5430, respectively. Additional relevant dataset accessions are provided together with their description in the Supplementary Material. Owing to human volunteer protection constraints, the sample metadata for the HIV cohorts will be provided upon request to HNRC: https://hnrp.hivresearch.ucsd.edu/index.php/hnrc-home.

## Code availability:

The code used for creating and implementing the CMMC enrichment workflow within the GNPS2 ecosystem is available at https://github.com/Wang-Bioinformatics-Lab/CMMC_GNPSNetwork_Enrichment_Workflow. The code used to create the CMMC-Dashboard MetaboApp is available at: https://github.com/wilhan-nunes/streamlit_CMMC_analysis-dashboard. The code used for dataset analyses can be found at: https://github.com/helenamrusso/CMMC-KB_manuscript.
